# Health-Related Composition and Bioactivity of an *Agave* Sap/Prickly Pear Juice Beverage

**DOI:** 10.3390/molecules29122742

**Published:** 2024-06-08

**Authors:** Luisa Fernanda Duque-Buitrago, Iraham Enrique Solórzano-Lugo, Marcela González-Vázquez, Cristian Jiménez-Martínez, María Antonia Hernández-Aguirre, Perla Osorio-Díaz, Georgina Calderón-Domínguez, Verónica Loera-Castañeda, Rosalva Mora-Escobedo

**Affiliations:** 1 Escuela Nacional de Ciencias Biológicas, Instituto Politécnico Nacional, Ciudad de México 07738, Mexico; luisaduquebuitrago@gmail.com (L.F.D.-B.); cjimenezh@ipn.mx (C.J.-M.); gcalderon@ipn.mx (G.C.-D.); 2 Escuela de Ingeniería de Alimentos, Universidad del Valle, Cali 76001, Colombia; 3 Instituto de Farmacobiología, Universidad de la Cañada, Teotitlán de Flores Magón 68540, Mexico; marcela.gonzalez@unca.edu.mx; 4 Centro de Desarrollo de Productos Bióticos, Instituto Politécnico Nacional, Yautepec 62731, Mexico; mhernandezag@ipn.mx (M.A.H.-A.); posorio@ipn.mx (P.O.-D.); 5 Centro Interdisciplinario de Investigación para el Desarrollo Regional Unidad Durango, Instituto Politécnico Nacional, Durango 34220, Mexico; vloera@ipn.mx

**Keywords:** *Agave* spp., *Opuntia* spp., aguamiel, cactus pear, gut fermentation, carbohydrates, phenolic compounds, short-chain fatty acids

## Abstract

In this study, a beverage made from a combination of *Agave* sap (AS) and prickly pear juice (PPJ) was analyzed for its nutrients and bioactive and potentially health-promoting compounds. The beverage was evaluated for its ability to act as an antioxidant, regulate glycemic properties, and undergo gut bacterial fermentation in vitro. The major mono- and oligosaccharides present in the beverage were galacturonic acid (217.74 ± 13.46 mg/100 mL), rhamnose (227.00 ± 1.58 mg/100 mL), and fructose (158.16 ± 8.86 mg/mL). The main phenolic compounds identified were protocatechuic acid (440.31 ± 3.06 mg/100 mL) and catechin (359.72 ± 7.56 mg/100 mL). It was observed that the beverage had a low glycemic index (<40) and could inhibit digestive carbohydrases. The combination of ingredients also helped to reduce gas production during AS fermentation from 56.77 cm^3^ to 15.67 cm^3^. The major SCFAs produced during fermentation were butyrate, acetate, and propionate, with valerate being produced only during the late fermentation of the AS. This beverage is rich in bioactive compounds, such as polyphenols and dietary fiber, which will bring health benefits when consumed.

## 1. Introduction

The functional food and beverage market has grown due to increased consumer awareness of health and wellness. Beverages made from the combinations of fruit juices and other foods are considered reliable sources of many biologically active compounds that can help in maintaining an active and healthy lifestyle, with ingredients focused on immunity such as prebiotics, antioxidants, and “superfruits” [1]. An important aspect of functional food development is the use of regional underutilized or neglected food resources to support food chain systems, promote sustainable development, conserve natural resources, and protect biodiversity [2]. *Agave* and *Opuntia* are unique resources found in xeric regions of Mexico and around the world.

One of the most rapidly evolving areas of functional food production is that which focuses on modulating the gut fermentation microbiota and promoting colonic health. The gut fermentation microbiota refers to the millions of microorganisms that symbiotically inhabit the human body and play a critical role in human health and disease [3]. A considerable proportion of bacterial metabolic processes that are beneficial to the host are involved in the extraction of energy through the fermentation of food waste (dietary fiber), particularly carbohydrates that are not digested in the upper gastrointestinal tract [4]. The bacterial gut fermentation of dietary fiber induces several changes in the metabolic environment of the intestinal lumen that may be beneficial to health; these changes include a decrease in pH, the softening of feces, and defense against pathogens [5,6].

The fermentation of dietary fiber produces short-chain fatty acids (SCFAs), including acetic, propionic, and butyric acids [7]. These SCFAs have several beneficial effects on the body and are responsible for most of the health benefits associated with fiber intake. They are rapidly absorbed in the colon and play a critical role in regulating gut motility, inflammation, and glucose levels [8]. Diverse types of dietary fiber have varying effects on different parts of the intestine, and it is important for prebiotics to stimulate bacterial activity consistently throughout the colon. Rapid fermentation is necessary because most absorption occurs in the proximal colon, but sustained fermentation is also beneficial in the distal region [9]. To achieve this, prebiotic blends or products with multiple sources of prebiotics can be used to modulate the microbiota and address the limitations of a single type of prebiotic [10].

Two traditional Mexican products that have potential as functional foods due to their prebiotic properties are *Agave* sap (AS) and prickly pear juice (PPJ). AS is a thick, sweet, and whitish liquid extracted from plants of the genus *Agave* (*Asparagaceae*), specifically species such as *A. americana*, *A. atrovirens*, *A. hookerii*, *A. inaequidens*, *A. mapisaga*, *A. marmorata*, and *A. salmiana* [11]. AS is traditionally consumed either fresh or fermented to produce a mildly alcoholic beverage called “pulque”. This beverage is composed of sugars, including fructooligosaccharides, which are considered to be bioactive compounds. These compounds are known for their proven activity as soluble and prebiotic fibers, as well as immunomodulators [12].

Prickly pears are the fruits of plants belonging to the genus *Opuntia* (*Cactaceae*). The most commonly cultivated species in Mexico for fruit production include *O. albicarpa*, *O. amyclaea*, *O. cochenillifera*, *O. ficus-indica*, *O. hyptiacantha*, *O. megacantha*, *O. robusta*, and *O. streptacantha* [13]. *Opuntia* is a genus that is known for its diverse profile of bioactive compounds, with kaempferol, isorhamnetin, and quercetin being the main flavonoids found across its species [14]. In addition, it has a high content of pectins and pectic polymers that are rich in galacturonic acid, as well as the residues of rhamnose, xylose, galactose, and arabinose. These compounds have prebiotic properties and are associated with different linkages between the residues [15].

The combination of AS and PPJ, each containing different bioactive compounds, offers a potential enhancement of their functional properties when used together rather than separately. Each ingredient is beneficial on its own; however, their combination can enhance the overall benefits through the addition of unique components and synergistic effects. The objective of this research was to evaluate the combined composition of a beverage made from AS and PPJ, assess its in vitro antioxidant capacity, analyze the in vitro intestinal fermentation of its indigestible fraction (dietary fiber), and subsequently measure the production of short-chain fatty acids.

## 2. Results

### 2.1. Composition of AS, PPJ, and the Beverage

Table 1 shows the proximal composition of the AS, PPJ, and beverage. The AS had a higher moisture content than the other samples, and the PPJ had a slightly higher ash content, but the difference was not statistically significant (*p* > 0.05). The protein content was not significantly different among the three products.

#### HPLC Quantification of the Monomers and Oligomers of the Carbohydrates of the AS, PPJ, and Beverage

Table 2 shows the results of the HPLC quantification of the different monomers and oligomers of carbohydrates. The main carbohydrates found in the AS were mannose, rhamnose, fructose, and galacturonic acid, with no detection of galactose. The PPJ had higher amounts of galacturonic acid, rhamnose, glucose, and galactose, with no detection of mannose. The AS contained statistically (*p* < 0.05) higher amounts of nystose and fructose than the PPJ. In the PPJ, nystose, glucose, galacturonic acid, and rhamnose were significantly higher (*p* < 0.05). The combined beverage showed significant differences in all the disaccharides and monosaccharides quantified compared to its individual ingredients.

### 2.2. Potential Health-Promoting Compounds of the AS, PPJ, and Combined Beverage

#### 2.2.1. Content of Total Bioactive Compounds in the AS, PPJ, and Beverage

Table 3 shows the data on the bioactive compounds in the beverage, assessed as the total phenolic content (TPC) and flavonoids. It is worth noting that the TPC content in the beverage increased significantly due to the contribution of the PPJ. The same trend was observed in the flavonoid content of the beverage. The PPJ had significantly higher levels of catechin (flavonoid) and protocatechuic acid (phenolic acid) but not gallic acid (phenolic acid), which did not differ significantly among the samples, or myricetin (flavonoid), which was not detectable in the PPJ. Furthermore, the beverage composed of the AS and PPJ showed statistically significant differences (*p* < 0.05) from the PPJ in terms of catechin, protocatechuic acid, and myricetin. Finally, the content of ascorbic acid (vitamin C) was similar in all three products.

#### 2.2.2. Antioxidant Capacity

An antioxidant is a molecule that is stable enough to donate an electron or proton to a free radical and neutralize it [16]. Table 4 presents the results of the determination of the iron chelation capacity (PFRAP) and antioxidant capacity measured via superoxide anion scavenging activity (SASA) and 2,2-azinobis (3-ethylbenzothiazoline-6-sulfonic acid) (ABTS) methods. All the determinations showed statistically significant differences among the samples. The AS showed the lowest antioxidant capacity, while the PPJ presented the highest value.

#### 2.2.3. Glycemic Regulatory Properties

Table 5 presents the functional properties of the glycemic index, as well as the in vitro inhibition of α-amylase and α-glucosidase. The glycemic index values of the samples in this study were below 40%, which classifies them as low-glycemic-index foods [17], with statistically significant differences among the samples (*p* > 0.05). The AS and the beverage demonstrated an inhibitory effect on α-glucosidase activity, but none of the samples showed an inhibitory effect on amylase activity.

### 2.3. In Vitro Gut Fermentation

Fermentation was conducted successfully, with all vials remaining under anaerobic conditions and metabolically active as determined using resazurin dye.

#### 2.3.1. Gas Production

**Figure 1:**
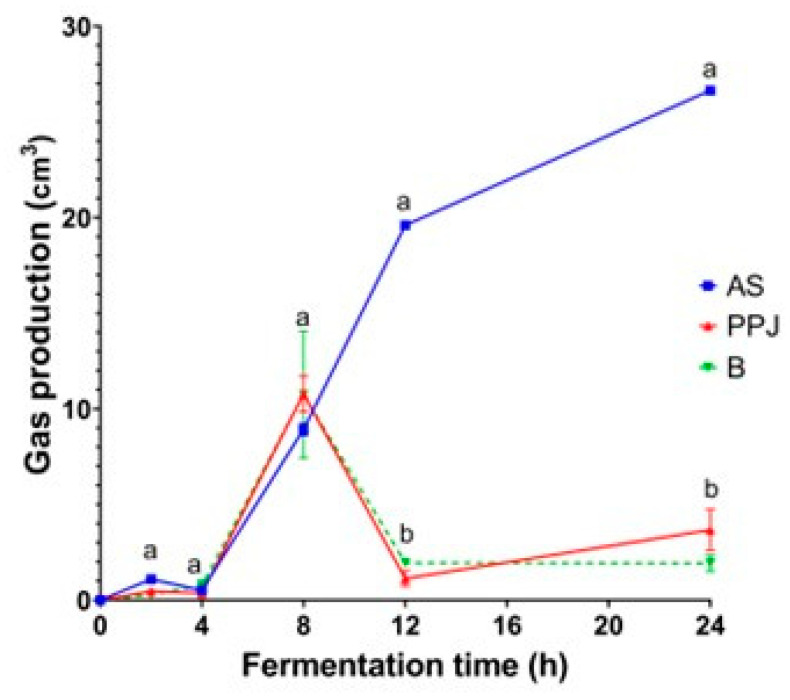
Gas production during in vitro gut fermentation. The presented values are means and standard deviation from three replicates. Different letters (a, b) show significant differences between the substrates at the same time points (*p* < 0.05). AS = *Agave* sap; PPJ = prickly pear juice; B = beverage.

Figure 1 depicts the volume of gas produced by the microbiota during in vitro fermentation. The maximum gas production in the PPJ occurred at 8 h, followed by a decrease associated with the complete fermentation of the substrate. The volume of gas in the fermentation of the PPJ was similar to that of the negative control (Supplementary Figure S1). The beverage also had the maximum volume of gas produced at 8 h, with values similar to the PPJ. During the first 4 h of the fermentation of all substrates, gas production was low and increased at similar levels after this period, with no significant differences between the substrates (*p* < 0.05). However, after 8 h, the gas production in the AS increased significantly up to 24 h. In comparison, the gas production in the PPJ and the beverage decreased at 12 h. The gas volumes maintained lower values until the end of the fermentation, with no significant differences between the PPJ and the beverage. This trend of a maximum value and a later decrease concerns the positive and negative controls (Supplementary Figure S1). The positive control reached a maximum point at 12 h with a later decrease. Conversely, the negative control maintained low gas volumes similar to those of the PPJ.

#### 2.3.2. Variation in pH

**Figure 2:**
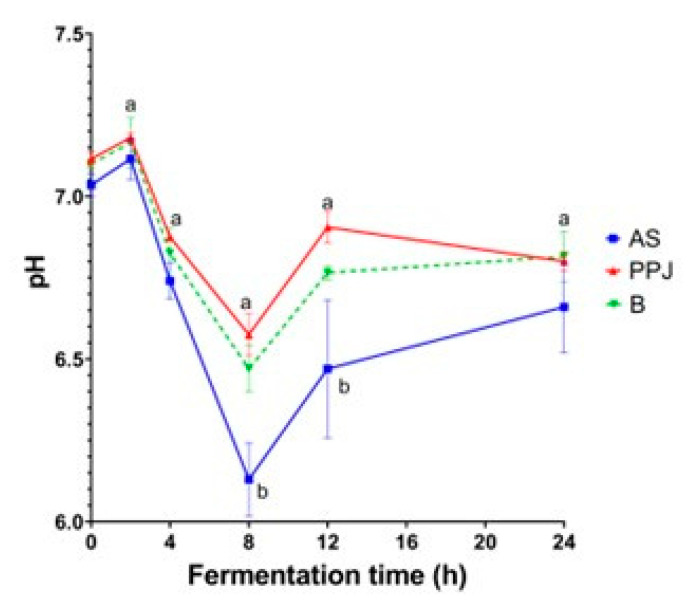
pH changes during in vitro gut fermentation. The presented values are means and standard deviation from three replicates. Different letters (a, b) show significant differences among the substrates at the same time points (*p* < 0.05). AS = *Agave* sap; PPJ = prickly pear juice; B = beverage.

Figure 2 shows the changes in the pH that occurred during the fermentation of the AS, PPJ, and beverage. Because of the production of SCFAs, the pH of the colon increased from 6.0 in the cecum to 6.7 in the distal colon. A further reduction in gut pH may be linked to inflammatory bowel diseases [18].

The pH levels of the samples remained stable at the beginning of the fermentation process, with no significant differences between them (*p* < 0.05). However, after 2 h, there was a slight increase. At 4 h, the pH decreased to values below 7, and there were no significant differences between the substrates. The lowest pH values were recorded at 8 h, with a significant difference between the AS substrate and the PPJ and beverage substrates. At 12 h, the pH stabilized, with a significant difference between the AS substrate and the other substrates. At the end of the fermentation process, all the substrates had similar pH values, with minimal changes between 12 and 24 h. Although the PPJ substrate had a higher pH tendency than the AS substrate, it still had a lower pH than the harmful control fermentation (Supplementary Figure S2). The minimum pH at 8 h, followed by stabilization, was consistent with the positive control pH.

**Figure 3:**
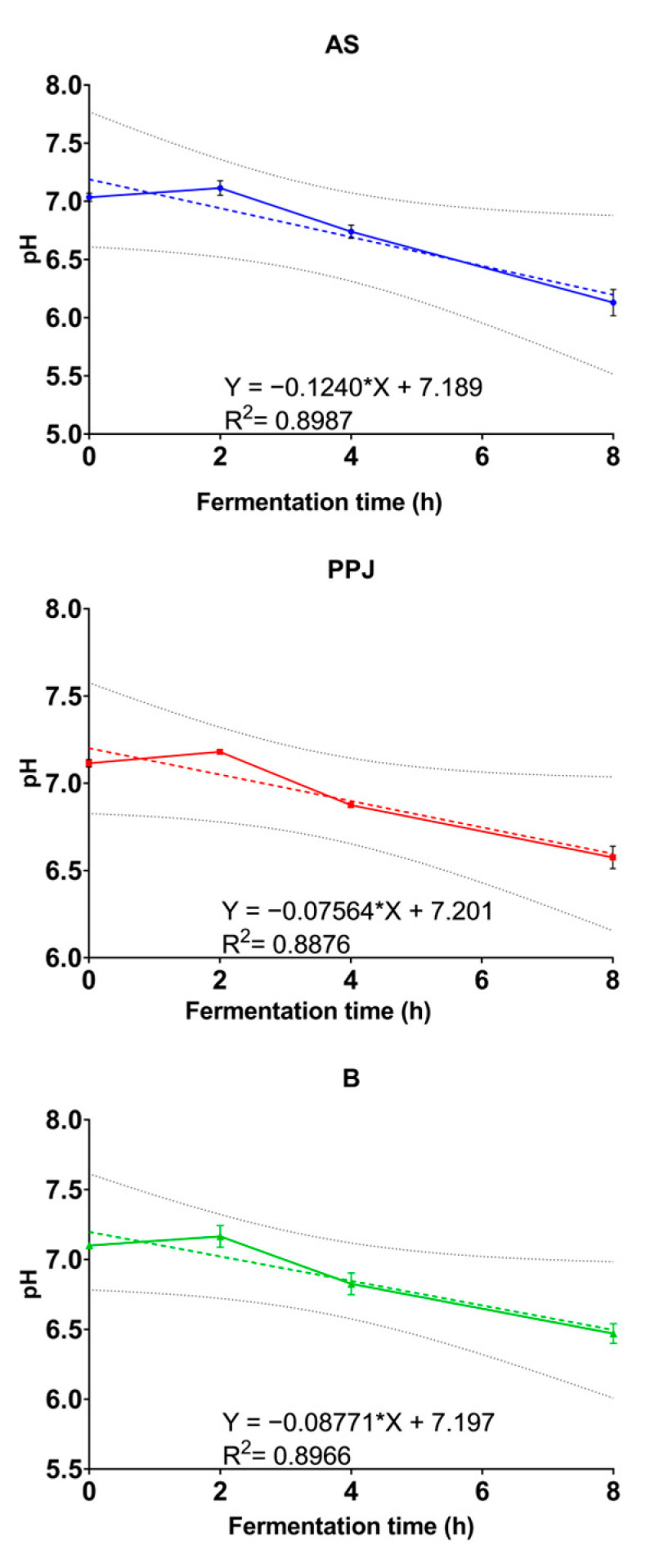
pH/fermentation rate time (h) change and fit of a linear regression model, where X is the slope of the linear equation. AS = *Agave* sap; PPJ = prickly pear juice; B = beverage.

Figure 3 shows the trend in the behavior of the AS, PPJ, and beverage samples during fermentation after linearizing the data. The results show that the pH of the medium decreased for all substrates, as confirmed by the negative slope obtained for each. The AS substrate had the most significant pH decrease in the shortest time, indicating that it was easily fermentable. However, the combination of the AS and PPJ in the beverage did not alter the fermentation characteristics of either one independently. It was expected that R^2^ would not be high since neither bacterial growth nor the accumulation of its metabolites follows linear growth.

#### 2.3.3. Short-Chain Fatty Acid Production

**Figure 4:**
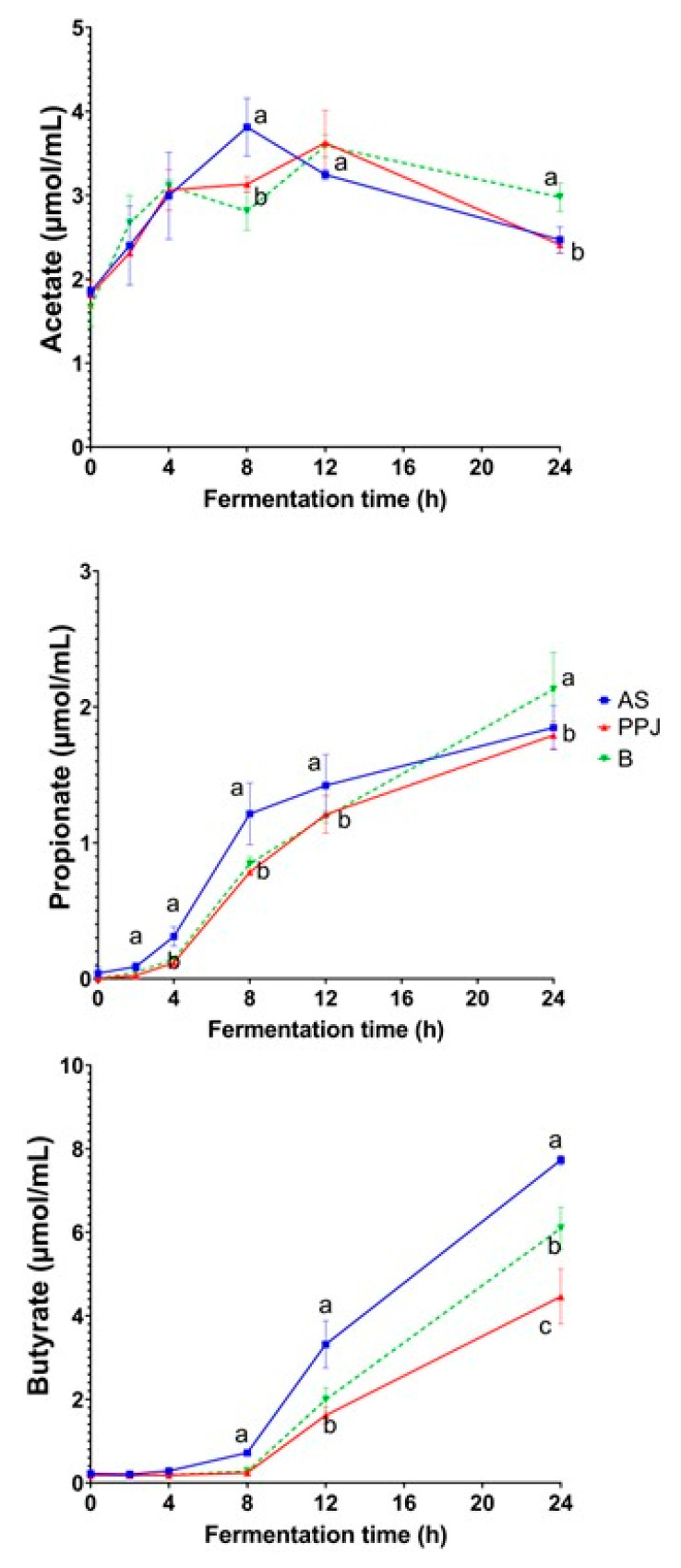
SCFA production during the in vitro fermentation process. The presented values are means and standard deviation from three replicates. Different letters (a, b, c) show significant differences between the substrates at the same time points (*p* < 0.05). AS = *Agave* sap; PPJ = prickly pear juice; B = beverage.

The changes in SCFAs (acetate, propionate, butyrate, and valerate) during the in vitro gut fermentation process are shown in Figure 4, while Supplementary Table S2 shows the total amount of SCFAs produced and the molar ratios after 24 h of in vitro gut fermentation for the AS, PPJ, and beverage. The initial concentration of SCFAs is related to the other fermentation by-products present in the donors’ feces. The production of SCFAs increased over time during the in vitro fermentation. The variation in SCFA production is due to the unique chemical properties of each compound and its compatibility with the bacteria present in the gut microbiota [19]; excessive gas production during the fermentation of prebiotics is considered undesirable. Regarding the fermentation process, the continuous increase in gas production during the fermentation of the AS shown in the analysis can be attributed to the fermentation of carbohydrates [20].

The concentration of acetate increased steadily from the start of fermentation up to 4 h, without any differences between the substrates. At 8 h, the fermentation reached the maximum concentration, with significant differences observed in the PPJ and the beverage. The production of acetate peaked at 12 h in both the PPJ and the beverage, and there were no significant differences between the substrates. However, between 12 and 24 h, the acetate concentration decreased across all the substrates. Notably, the fermentation of the beverage significantly differed from that of the AS and PPJ during this time.

The concentration of propionate steadily increased from the start of fermentation up to 24 h in all the substrates. Between 4 and 12 h, the AS fermentation produced more propionate than the PPJ and beverage fermentation, and there were significant differences. By the 24 h mark, the propionate concentration in the beverage surpassed that in the AS and PPJ, demonstrating a significant difference. This highlights an interaction in the combination of the AS and PPJ. Notably, propionate was the third SCFA produced in all the substrates.

Butyrate was not initially present, but its production began 4 h after the fermentation of the substrates. It continued up to 8 h in the AS, PPJ, and beverage, with no significant differences in concentration. At 12 h, the butyrate production increased, with the AS showing the highest accumulation values, significantly different from those of the other substrates. Its production continued for up to 24 h, with significant differences between all the substrates. The AS had the highest production, followed by the beverage and PPJ.

Isobutyrate and isovalerate were not quantifiable at any fermentation time. The molar ratios of all the SCFAs produced in the in vitro gut fermentation of the AS, PPJ, and beverage after 24 h are shown in Supplementary Table S2. The AS fermentation produced the highest amount of propionate and significantly higher amounts of acetate and butyrate. The fermentation of the PPJ presented the highest propionate ratio of all substrates but with no significant differences. The fermentation process of the beverage showed butyrate and propionate increases similar to those of the AS. Still, the acetate molar ratio was similar to that of the PPJ and significantly different from that of the AS.

## 3. Discussion

### 3.1. Composition of the AS, PPJ, and Beverage

Table 1 shows that the ash content was the highest in the PPJ. Prickly pears are rich in essential elements, such as potassium, calcium, and magnesium, and they contain trace minerals, such as manganese and copper [21]. These minerals are essential for various cellular processes that are critical for overall health, cardiovascular and metabolic well-being, and endogenous antioxidant mechanisms [22,23].

#### 3.1.1. HPLC Quantification of the Monomers and Oligomers of the Carbohydrates of AS, PPJ, and Beverage

The concentrations of monosaccharides, such as glucose, fructose, galactose, rhamnose, arabinose, and galacturonic acid, found in this study are consistent with those reported by El Kossori et al. (1998) [24] and Habibi et al. (2004) [25]. These compounds are responsible for the sweet taste of AS, PPJ, and the beverages derived from them. Besides these, other carbohydrate monomers are found in AS, such as nystose [26]. In PPJ, galacturonic acid, mannose, and galactose are present. Notably, the combined beverage has less galacturonic acid than PPJ.

AS and PPJ are known for their unique properties. AS contains fructooligosaccharides, which are derived from crassulacean acid metabolism. These fructooligosaccharides accumulate highly branched fructans called agavins, which serve as reserve carbohydrates [12]. Prickly pears and the cladodes of *Opuntia* are also compelling due to their accumulation of pectic substances and mucilage [15]. Both the AS and PPJ are rich in carbohydrates, making them sources of soluble fiber. This fiber classifies them as prebiotics, which are non-digestible food ingredients that have a beneficial effect on the host by selectively stimulating the growth or activity of specific bacteria in the colon. Thus, dietary fiber interventions may provide a solution to reducing the societal burden of gut diseases, such as obesity and metabolic syndrome, and enhancing the host’s health and well-being [27]. This is due to the microbiota that is favored by the consumption of dietary fiber.

#### 3.1.2. Content of Total Bioactive Compounds in AS, PPJ, and Beverage

Phenolic compounds are of great scientific interest due to their diverse biological activities and potential functional properties. They are widely available and present in our diet. The most well-known and widely distributed phenolic compounds include anthocyanins, coumarins, flavonoids, and phenolic acids [28]. Phenolic compounds play a critical role in mitigating oxidative stress, reducing cellular damage caused by free radicals, enhancing the body’s redox defense mechanisms, and reducing the risk of developing diseases associated with free radical damage. Such diseases include inflammation, cancer, cardiovascular disease, and neurodegenerative disorders. The presence of phenolic compounds is also a significant factor in the growing demand for these substances [29].

As shown in Table 2, the AS, PPJ, and beverage all contained significant amounts of total phenols (TPCs) and flavonoids, with the PPJ having the highest levels. Several specific compounds were identified in the experimental AS samples, such as ascorbic acid, catechin, protocatechuic acid, and myricetin, which together contribute to a total quantified content of 79.97 ± 6.79 mg gallic acid equivalents per 100 mL. These results indicate the presence of significant antioxidant and bioactive compounds in the AS, highlighting its potential for various health-related applications, as phenolic compounds have been reported to have diverse anti-inflammatory and protective effects [30,31].

The analysis of the PPJ revealed a rich variety and concentration of phenolic compounds (62.415 ± 0.24 mg GAE per 100 mL). The flavonoid content, which was measured at 15.92 ± 1.85 mg CE per 100 mL, supports the presence of flavonoids such as quercetin and kaempferol. The presence of significant amounts of catechin (63.53 ± 3.20 mg CE per 100 mL), protocatechuic acid (84.43 ± 0.75 mg per 100 mL), and gallic acid (1.23 ± 0.02 mg per 100 mL) confirms the diversity of phenolic compounds. Although myricetin was not detected, the total quantified phenolic and antioxidant compound content of 153.78 ± 4.1 mg per 100 mL highlights the potential health benefits of the PPJ. These findings underscore its role as a valuable natural product with antioxidant and health-promoting properties. El-Hawary et al. (2020) reported that the prickly pear exhibited a rich array of chemical constituents, including steroidal saponins, flavonoids, homo-isoflavones, cinnamic acid derivatives, and fatty acids, providing valuable insights into its metabolic composition [32]. They found that all the tested extracts exhibited substantial antioxidant activities in vitro and neuroprotective potential under AlCl_3_-induced Alzheimer’s conditions.

Marquez-Lemus et al., 2022, showed that the red and purple varieties of prickly pear have a significantly higher total phenolic content (TPC) than the lighter-colored varieties such as white and yellow, and they stated that the flavonoid content in PPJ is similar to that found in certain Mexican prickly pear varieties [33,34]. However, the composition and relative proportions of different flavonoids can vary significantly depending on the specific variety being studied. In this work, the analysis revealed the presence of primary flavonoids such as quercetin, iso-rhamnetin, kaempferol, myricetin, and luteolin.

Phenolic compounds are of particular interest as possible functional ingredients due to their ubiquitous availability, abundant resources in our diet, and diverse biological activities [28]. Protocatechuic acid and catechin were the major phenolic compounds in the AS and PPJ. Protocatechuic acid is a primary metabolite in the configuration of complex polyphenols. Interestingly, the beverage presented a combination of the phenolic compounds found in the AS and PPJ, making it possible to say that it can be offered as a functional drink, which will help to control diet-related diseases.

### 3.2. Potential Health-Promoting Compounds of the AS, PPJ, and Combined Beverage

#### 3.2.1. Antioxidant Capacity

The evaluation of antioxidants often involves multiple assays using different methods. In this case, this included ABTS and PFRAP assays based on electron transfer, the SASA method based on hydrogen atom transfer, and PFRAP based on transition metal chelation [16,35]. The significant differences in the antioxidant and iron chelation capacities among the samples are shown in Table 4.

The variation in the antioxidant capacity values observed in the AS can be attributed to the inherent variability in the sap samples influenced by climatic conditions, as AS is collected outdoors, and environmental factors such as rain or drought can significantly affect the concentration of antioxidant compounds. For instance, Romero-López et al. (2015) [36] reported an antioxidant capacity of 8.88 μmol TE in AS using the ABTS method, while other samples of AS yielded values ranging from 8.5 to 10.7 mg TE [37].

The incorporation of foods rich in antioxidants, such as the PPJ and beverage, offers numerous health benefits, including a reduced risk of chronic diseases and improved well-being by neutralizing harmful free radicals, thereby reducing oxidative stress [38,39,40]. Although human studies do not show a clear increase in serum antioxidant capacity, significant differences are found in parameters related to oxidative stress. Research consistently indicates that the consumption of vegetable and fruit juices is associated with a decreased incidence of chronic diseases due to the high bio-accessibility of bioactive compounds, particularly phenolic compounds [38,41]. The different measurements carried out in this research provide a broader picture of the antioxidant capacity of the combined beverage and its components that combat oxidative stress through different chemical mechanisms.

#### 3.2.2. Glycemic Regulatory Properties

Inhibiting α-amylase, a key enzyme in starch and glycogen digestion, is increasingly acknowledged as a therapeutic approach for managing conditions associated with carbohydrate uptake dysfunction, such as diabetes, obesity, dental caries, and periodontal diseases [42,43]. This strategy, when combined with a low-glycemic-index diet, can significantly aid in regulating blood glucose levels. Among the array of metabolites found in plant foods, dietary polyphenols stand out for their therapeutic potential in the prevention and management of diabetes. This recognition stems from their capacity to safeguard pancreatic cells against glucose toxicity, their anti-inflammatory and antioxidant attributes, and their ability to suppress digestive enzymes while inhibiting starch digestion [44,45].

In this study, the inhibitory potential of the AS, PPJ, and beverage on α-amylase and α-glucosidase enzyme activities was investigated. No inhibitory effects were observed on α-amylase. However, in the α-glucosidase enzyme inhibition assay, the AS and beverage demonstrated significant inhibition. Previous studies have reported the inhibitory activity of different *Agave* syrups, with values of 21.19% and 24.8% for *A. atrovirens* var. pulquero [46], as well as inhibition by prickly pear products [31,47]. α-Glucosidase inhibitors play a key role in attenuating intestinal carbohydrate absorption and mitigating postprandial glucose spikes. Although they do not completely inhibit carbohydrate absorption, they significantly contribute to the reduction in postprandial insulin and glucose peaks [48,49].

The identification of inhibitory activity in the AS but not in the PPJ suggests that the metabolites differentially present in the AS may generate this activity. Of all the metabolites screened, only myricetin was identified as differentially present in the AS. Myricetin was previously reported to have significant inhibitory effects in a dose-dependent manner, with an IC_50_ of 41.14 ± 2.52 against α-glucosidase [50]. Molecular docking analyses showed that myricetin formed nine hydrogen bonds with α-glucosidase. In a study of α-glucosidase inhibition with various flavonoids, including myricetin, quercetin, and catechins, myricetin showed the highest inhibitory activity, along with catechins. Both myricetin and catechin are reported to be competitive inhibitors that bind to the active site of the enzyme through hydrogen bonding [51].

Carbohydrase inhibition by plant foods has been attributed to various plant metabolites, including phenols, saponins, fatty acids, sterols, triterpenes, alkaloids, anthraquinones, and tannins, which may contribute to α-glucosidase inhibition [52]. The present study provides further evidence of the potential of plant metabolites to inhibit α-glucosidase activity, as evidenced by the significant inhibitory effects of the AS and beverage. Interestingly, while no inhibitory effects on α-amylase were observed, the selective identification of myricetin in the AS suggests that it has a specific role in α-glucosidase inhibition. The inhibitory effects of myricetin, together with its ability to form hydrogen bonds with α-glucosidase, highlight its potential as a competitive inhibitor. These findings contribute to our understanding of the therapeutic potential of plant-derived compounds in the management of conditions associated with carbohydrate uptake dysfunction, such as diabetes and obesity.

The results of this work are important because patients with type 2 diabetes have an inadequate insulin response after a meal, leading to challenges in glucose regulation. Therefore, slowing glucose absorption is important to normalize the pancreatic insulin response and mitigate postprandial hyperglycemia, a condition strongly associated with cardiovascular mortality [53].

#### 3.2.3. Interactions of Bioactive Compounds

In the present study, several bioactive compounds were quantified, including ascorbic acid and protocatechuic acid, both of which contain two hydroxyl groups (-OH), and catechin, gallic acid, and myricetin, which contain three hydroxyl groups. Despite the expectation of a stronger correlation between gallic acid and myricetin due to their similar number of hydroxyl groups, no significant trends were observed. In addition to hydroxyl groups, it is important to consider the concentrations of each ingredient in the beverage. This comprehensive assessment suggests a potential synergistic effect between all the antioxidant compounds present [42].

A multivariate Pearson correlation analysis via HPLC (see Supplementary Table S1) indicated significant positive correlations between the total phenolic content (TPC) and flavonoid content, PFRAP, SASA, and ABTS. The flavonoid content also demonstrated positive correlations with PFRAP, SASA, and ABTS, with SASA exhibiting the highest linear correlation (R^2^ = 0.973) with flavonoids. Furthermore, PFRAP showed positive correlations with SASA and ABTS, while SASA exhibited a negative correlation with ABTS. The reducing power of the extracts from the prickly pear pulp is statistically linked to the phenolic compound concentration. Conversely, the AS displays a comparatively lower antioxidant capacity, suggesting a potential need for additional dietary supplementation for enhanced antioxidant protection. However, combining the PPJ and AS offers an alternative to mitigate AS’s low antioxidant capacity. This effect could be mediated by the modulation of antioxidant enzyme activities and high total phenolic content. The beverage with the AS and PPJ could then be used as a dietary supplement to improve oxidative status.

### 3.3. In Vitro Gut Fermentation

#### 3.3.1. Gas Production

The microbiota, including saccharolytic species, produce gases such as carbon dioxide, methane, and hydrogen as by-products of their metabolic activities [54]. This gas production is particularly high when rapidly fermentable dietary fibers are consumed, leading to gastrointestinal symptoms such as increased flatulence and bloating [19]. Therefore, excessive gas production during prebiotic fermentation is considered undesirable. The continuous increase in gas production during the fermentation of the AS up to 24 h (Figure 1) can be attributed to the fermentation of small intestinal undigested carbohydrates such as oligosaccharides, sugars, and galacturonic acid [19]. The continuous and steady gas production during the fermentation of the AS for 24 h is more advantageous than that of other fructose polymers, which show exaggerated gas production in the first part of fermentation. The amount and structure of oligosaccharides in agave could be induced to increase gas production compared to that of other fructose polymers.

Meanwhile, the fermentation of the PPJ and the resulting beverage showed a pattern of gas production with the maximum output occurring around 8 h, followed by a decline as the substrates fully fermented. This could be due to the trapping of molecules in the food matrix, which then could have affected the digestibility and fermentability of the PPJ and beverage. This occurs due to the physical obstruction of molecules, preventing them from reaching the active sites of enzymes [55]. The pectic substances and mucilage characteristics unique to the genus *Opuntia* may contribute to gas production patterns. For beverage fermentation, a reduction in gas production is usually desirable if it does not interfere with the production of other important by-products such as SCFAs.

#### 3.3.2. Variation in pH

The changes that occurred during the fermentation of the AS, PPJ, and beverage can be seen in Figure 2. The initial pH was similar for the three samples, with a slight increase after 2 h, followed by a drop after 4 h of fermentation. Within the first 4 h of the gut fermentation of pectic polysaccharides, a rapid change in pH occurs as reported by Cantu-Jungles et al. (2019) and Gulfi et al. (2005, 2006) [56,57,58]. The maximum drop occurred after 8 h, with statistical differences between the AS, PPJ, and beverage (*p <* 0.05); after 12 h, the pH stabilized across all the substrates. This pH pattern was similar to that of the positive control (Supplementary Figure S2).

The AS contains carbohydrates with a low DP, typically consisting of two to nine monomers [59]; the short size of these molecules is expected to facilitate fermentation. During the fermentation process of the AS, the pH levels were observed to be the lowest at the 8 h mark, suggesting a continuous fermentation process lasting more than 12 h. This sustained fermentation can be beneficial for the distal colon. The fermentation of a substrate in the gut results in a reduction in pH, which has a positive impact on gut health. Lower pH levels in the colon have been shown to reduce the formation of secondary bile acids while also increasing the availability of calcium [60] and providing other beneficial physiological effects such as a reduction in inflammation, especially when combined with probiotics [61]. The fructan fractions possess prebiotic activities and have beneficial effects via the inhibition of intestinal pathogen growth. García-Gamboa et al. (2020) reported that, on day 4 of the administration of fructans with a low molecular weight, significant increases in *Lactobacillus* spp., *Clostridium* spp., and *Salmonella* spp. (*p* < 0.05) were observed in the transverse colon section, whereas by day 9 of administration, *Lactobacillus* spp. had increased in the ascending colon, *Bifidobacterium* spp. had increased in the descending colon, and *Salmonella* spp. had decreased in the transverse colon [62].

The pH changes observed are linked to the specific SCFAs produced during gut fermentation, with each SCFA having a distinct and variable impact on pH values. For example, the SCFA acetate has the lowest pKa value of 4.75, followed by valerate (4.80), butyrate (4.81), and propionate (4.88) [63].

#### 3.3.3. Short-Chain Fatty Acid Production

In relation to the production of SCFAs during the fermentation of the AS, PPJ, and combined beverage, it is important to note that acetate was consistently generated across all the substrates examined. This observation is supported by the well-known link between acetate production and the fermentation of sugars such as glucose, galactose, mannose, and xylose [9]. The samples analyzed were expected to exhibit acetate production derived from the bacterial fermentation of the glucose, galactose, and xylose monomers identified and quantified in the samples (Supplementary Table S2). Acetate is a primary fermentation product for many anaerobic bacteria in the intestine, and, thus, various dietary factors can influence acetate production by the gut microbiota [64]. Acetate can enter the portal circulation, and it seems to play a role in lipid and cholesterol synthesis in the liver, as well as providing energy through the oxidation of residual acetate in muscle cells, which can improve overall gut health [60].

Propionate production is primarily linked to the fermentation of fructose, arabinose, and other ketoses [9]. As shown in Figure 4, the total production of propionate in the fermentation of the AS and the beverage is higher than that in the fermentation of the PPJ. Regarding the total output, propionate was expected to be higher, as FOS fermentation allows for its depolymerization to increase the content of fructose monomers present in nystose, ketose, and sucrose. Louis and Flint (2017) provided an overview of the metabolic pathways utilized by gut microbes to produce propionate from dietary carbohydrates [64]. The dominant bacterial species detected in the fecal samples of human subjects were the phylum of Firmicutes, families *Lachnospiraceae*, *Ruminococcaceae*, and *Erysipelotrichaceae*.

The liver plays a crucial role in the metabolism of propionate, which is associated with numerous health benefits, including immune system support, cholesterol metabolism, satiety regulation, and gut function [65]. Previous studies have found that inulin and FOS fermentation produce higher amounts of propionate with higher polymerization [66,67]. Agave fructans with a higher DP have been shown to produce lower amounts of propionate than FOS [67]. The propionate concentration in fructans fermentation is lower than the acetate and butyrate concentrations, with comparable results for *Agave* fructans.

The most important SCFA produced in the AS, PPJ, and beverage was butyrate. This strongly suggests that butyrate production is determined by the supply of non-digestible carbohydrates [64]. The production of SCFAs can be used to predict the fermentation profile of different fermentable products or ingredients. Hernot et al. (2009) found a higher proportion of butyrate for medium-chain FOS with the molar ratios of 55:15:30 and inulin with the molar ratios of 45:13:42 at 12 h [67]. Stewart et al. (2008) reported even higher proportions of butyrate for different FOS and inulin substrates, with the molar ratio ranges of 58–63:7–8:29–35 for FOS and 58–67:7–10:24–35 for inulin substrates [68]. These ratios indicate that all the substrates favored butyrate production over propionate production, like the molar ratios found in the fermentation of the AS, PPJ, and beverage (Supplementary Table S2). The production of SCFAs can be used to predict the fermentation profile of different fermentable products or ingredients.

Gut bacteria that degrade dietary fiber target specific bonds in certain types of polymers, but these primary degraders are not butyrate producers themselves [69]. The activities of secondary bacterial fermenters are essential to stimulate butyrate production. Butyrate production used the succinate pathway, with dominant bacterial species such as *Bacteroides uniformis*, *Bacteroides vulgatus*, *Prevotela copri*, and *Alistipis putredunis* [64]. Butyrate is an essential energy source for the gut mucosa; helps to protect against colorectal cancer; and has several physiological effects related to inflammation, the immune system, oxidative stress, gut barrier function, satiety, and insulin sensitivity [60,65].

During the fermentation of branched-chain amino acids by certain genera such as *Bacteroides* and *Clostridium*, valeric acid, isovaleric acid, and isobutyrate, collectively referred to as branched-chain SCFAs (BSCFAs), are synthesized [70,71]. However, the concentrations of isobutyrate and isovalerate remained undetectable throughout the duration of the fermentation process in the AS, PPJ, and beverage. Finally, it is important to note that carbohydrate fermentation could be a beneficial process in the large gut, because the growth of saccharolytic bacteria stimulates their requirements for toxic products associated with putrefaction for incorporation into cellular proteins, thereby protecting the host.

## 4. Materials and Methods

### 4.1. Collection

The sap was obtained from a traditional backyard plantation in the municipality of Contepec, Michoacán, Mexico (coordinates: 19°57′18″ N 100°12′44″ W). The plant, traditionally identified as “Maguey Manso”, is a species of Agave plant characterized by its considerable size, reaching up to 2 m in height, which facilitates its direct identification. This plant is related to the species Agave atrovirens [72,73]. It was then filtered and packaged in 250 mL sterilized glass-topped metal bottles. The sap was pasteurized at 121 °C and 15 psi for 10 min to prevent its characteristic spontaneous fermentation.

A juice was made using *Opuntia* sp. fruits, commonly known as “red cactus pear,” purchased from a local market in Mexico City. The fruits were washed and peeled, and the juice was obtained by putting the pulp in an industrial fruit pulper (Bertuzzi, Milan, Italy). A beverage, which was a combination of pasteurized AS and PPJ, was formulated using an optimized recipe of 60% pasteurized AS and 40% PPJ. The AS, PPJ, and combined beverage were pasteurized at 91 °C for 7 min. All the tests were carried out on the pasteurized AS, PPJ, and formulated beverage.

### 4.2. Composition

The proximal composition of the AS, PPJ, and beverage was determined using the AACC (2000) methods for moisture content (method 44-15), protein content (method 46-13), and ashes (method 08-01) [74].

### 4.3. Free Carbohydrate Quantification

The samples underwent centrifugation (using Velocity14 centrifuge, Metrix, Mexico City. Mexico) at 4025× *g* for 5 min. The resulting supernatant was collected and filtered (Millex^®^ 0.2 μm nylon filters, Millipore Corporation, Burlington, MA, USA). High-resolution liquid chromatography (HPLC) was used to quantify free carbohydrates according to the manufacturer’s application notes [75]. The chromatographic equipment consisted of a quaternary pump 1200 Infinity System (Agilent, Santa Clara, CA, USA) and a refractive index detector (RID, 1260, Agilent, USA). For the detection of nystose, ketose, saccharose, glucose, and fructose, a Hamilton Ca column (7.8 × 305 mm, 8 79436, Hamilton, Reno, NV, USA) was used. The mobile phase was water at a flow rate of 0.4 mL/min, with an injection volume of 10 μL, column temperature of 85 °C, and detector temperature of 55 °C. To detect glucuronic acid, galacturonic acid, mannose, galactose, xylose, and rhamnose, an Agilent Hi-Plex column H (7.7 × 300 mm, 8 μm, PL1170—6830, Agilent, Santa Clara, CA, USA) was used. The mobile phase was H_2_SO_4_ (0.01 M) at a flow rate of 0.4 mL/min and with an injection volume of 10 μL, and the column and detector temperatures were 55 °C [75]. Carbohydrates were identified and quantified using a standard curve prepared in advance with each of the selected carbohydrates (Supplementary Material S1).

### 4.4. Bioactive Compounds Analysis of the AS, PPJ, and Beverage

For the determination of the bioactive compounds of the AS, PPJ, and beverage, the samples were centrifuged at 4025× *g* (centrifuge Velocity14, Metrix, México) for 5 min, and the supernatant was collected and filtered (Millex^®^, 0.2 μm nylon filters, Millipore Corporation, USA).

#### 4.4.1. Total Phenolic Compounds and Flavonoids

The total phenolic compounds (TPCs) were measured using the Folin/Ciocalteau method, while the flavonoid content was assessed using an aluminum chloride colorimetric assay [76]. All spectrophotometric analyses were carried out using a microplate reader, Multiskan Go (Thermo, Waltham, MA, USA). To quantify the TPCs, a standard curve was created with gallic acid, with five calibration points ranging from 0.02 to 0.14 mg gallic acid/mL. The formula used for the curve was y = 11.824x − 0.0296, with an R^2^ value of 0.99. The results are reported as mg gallic acid equivalents/mL (AGE/mL) [77]. The flavonoid quantification was carried out using a standard curve prepared with quercetin, with five calibration points of 0, 50, 100, 150, 200, 250, and 300 µg of quercetin/mL (y = 0.0045x + 00.085; R^2^ = 0.99). The results are reported as µg of quercetin equivalents/mL (QE/mL) [78].

#### 4.4.2. Identification and Quantification of Individual Bioactive Compounds

Bioactive compounds were identified and quantified using high-performance liquid chromatography (HPLC). The chromatographic system utilized a quaternary pump (1200 Infinity System, Agilent, USA), along with a diode-array detector (DAD, 1260, Agilent, USA) and a ZORBAX Eclipse Plus C18 column (4.6 × 250 mm, 5 μm, 959990-902, Agilent, USA). The column and detector were both set to a working temperature of 35 °C. The mobile phase consisted of phosphoric acid 0.5% (A) and methanol (B) in a 40:60 ratio, with an isocratic flow rate of 1 mL/min [79]. The quantification of the bioactive compounds (rutin, gallic acid, catechin, caffeic acid, quercetin, p-coumaric acid, pelargonidin, ferulic acid, protocatechuic acid, ascorbic acid, myricetin, and chlorogenic acid) was accomplished using standard curves that had been previously prepared with each of the selected analytical standards (Supplementary Material S1).

### 4.5. Health-Related Biofunctionality

#### 4.5.1. Antioxidant Capacity

The AS, PPJ, and beverage samples were pasteurized and then centrifuged at 4025× *g* (centrifuge Velocity14, Metrix, México). The liquid portion (supernatant) was collected and tested for ABTS radical scavenging activity [80]. The results are expressed as mM Trolox equivalents (TE)/mL based on a Trolox calibration curve, with six points ranging from 0 to 250 mM Trolox. The calibration curve equation was y = −0.0025x + 0.703, with an R^2^ value of 0.99. Additionally, the samples were tested for superoxide anion scavenging activity (SASA) [81] and for iron chelation capacity with the potassium ferricyanide reducing power assay (PFRAP) [82]. The results of these tests are presented as a percentage of change in absorbance compared to a solution of gallic acid (0.05 M).

#### 4.5.2. Glycemic Index

The glycemic index was determined using the method proposed by Goñi et al. (1998) [83], with some modifications. The samples were frozen in 100 mL lyophilizer wide-mouth flasks (Labconco, Kansas, MO, USA) at −80 °C in an ultra-cold freezer (Esco Life Sciences, Singapore) and lyophilized in a freeze-dryer (FreeZone 2.5, Labconco, Kansas, MO, USA) at −40 °C and 0.2 mbar for approximately 24 h. First, 10 mg of a lyophilized sample was mixed with 2 mL HCl-KCl buffer (pH 1.5) and 0.04 mL pepsin solution. The mixture was then incubated in a shaking water bath (Dual Action Shaker and Water bath, PolyScience, Niles, IL, USA) at 40 °C for 1 h. After a period of incubation, a mixture of 5 mL Tris-maleate buffer (pH 6.9) and 1 mL α-amylase solution was added to the sample. The resulting solution was then incubated at 37 °C for 180 min. At 30 min intervals, 0.5 mL aliquots were taken from the mixture, and the reaction was stopped by subjecting the samples to thermal shock at 100 °C. The concentration of released glucose was measured using a glucose oxidase–peroxidase kit (GAGO20, Sigma-Aldrich Inc., Waltham, MA, USA). The in vitro digestion kinetics were determined according to the method proposed by González-Vázquez et al. (2022) [84].

#### 4.5.3. Enzymatic Inhibition of Carbohydrases

The enzymatic inhibition of two carbohydrases by the AS, PPJ, and beverage was performed with the original samples and with aqueous and ethereal extracts of the samples. To obtain the extracts of the samples, a volume of 50 mL was measured and deposited in an amber container with an equal volume of ether. It was left for 20 h in agitation, and then it was placed in a separation funnel to obtain the aqueous phase. The ethereal phase was deposited in an amber container, and a volume of 50 mL of distilled water was added to the ethereal extracts and placed in an extraction hood until the ether evaporated. Finally, the extracts (ethereal and aqueous) and original samples were subjected to centrifugation at 1792× *g* (centrifuge Velocity14, rotor 13, 10 cm radius, Metrix, México) for 10 min, and the supernatant was recovered for use in the enzyme inhibition tests.

To determine the enzymatic inhibition of α-amylase, 250 µL of the filtered samples and 250 µL of a solution of α-amylase (0.5 mg/mL) in sodium phosphate buffer (0.02 M, pH 6.9) were added and incubated at 25 °C for 10 min. Subsequently, 250 µL of 1% of starch solution in sodium phosphate buffer (0.02 M, pH 6.9) was added and incubated at 25 °C for 10 min, and, finally, 500 µL of dinitro salicylic acid reagent was added. The reaction mixture was diluted with 5 mL of distilled water, and the absorbance was read at 540 nm in a microplate reader (Multiskan Go, Thermo Fisher Scientific, USA) [48]. To determine the enzymatic inhibition of α-glucosidase directly in a 96-well microplate, 50 µL of sample and 100 µL of α-glucosidase solution (67 U) prepared in phosphate buffer (0.1 M pH 6.9) were incubated at 25 °C for 10 min. Subsequently, 100 µL of a 0.1 M solution of 4-Nitrophenyl-β-D- glucopyranoside diluted in phosphate buffer (0.1 M pH 6.9) was added and incubated at 25 °C for 30 min. The absorbance was read at 405 nm at the beginning and at the end of the 30 min incubation (Multiskan Go, Thermo Fisher Scientific, USA) [85].

### 4.6. In Vitro Gut Fermentation

Undigestible residue obtention: In the simulation of gut fermentation, foods must undergo digestion to ensure that the microbiota uses the materials that would reach the colon and not the free carbohydrates in the raw material. The AS, PPJ, and combined beverage were obtained following the standardized protocol developed by the INFOGEST research network [86]. The protocol consists of simulating the digestive process through the addition of salts and enzymes characteristic of the oral, gastric, and duodenal digestion (KCl, NaHCO_3_, MgCl_2_·(H_2_O)_6_, CaCl_2_·2(H_2_O), NaCl, and NaOH [Merck, Darmstadt, He, Germany]; KH_2_PO_4_ and HCl [J.T.Baker, Radnor, PA, USA]; human salivary α-amylase, porcine pepsin, and porcine pancreatin [Sigma-Aldrich, St. Louis, MO, USA]; and bovine bile [BioBasic, Markham, ON, Canada]) at 37 °C during orbital agitation at 100 rpm in an incubator shaker (Barnstead Lab-line, Model SHKA4000, Dubuque, IA, USA).

The chyle obtained from all simulated digestions was placed in a beaker with dialysis tubes with a nominal pore size cutoff of 12,000 Da (Sigma-Aldrich) and filled with 1 M NaHCO_3_ to filter out low-molecular-weight molecules that could be absorbed during the digestion process. The remaining chyle, without the dialyzed fraction, was placed in 100 mL lyophilizer wide-mouth flasks, frozen at −80 °C in an ultra-cold freezer (Esco Life Sciences, Singapore), and lyophilized in a freeze-dryer (MicroModulyo 1.5 L Freeze Dry, Thermo Fisher Scientific, Waltham, MA, USA) at −40 °C and 0.1 mbar for approximately 48 h. The lyophilized residue on the beakers after dialysis was considered non-digestible residue. The method proposed by Goñi and Martín-Carrón (1998) was followed for the in vitro gut fermentation setup; preparation of the medium, inoculum, and sample; and treatment [83].

Fermentation medium: A total of 2.5 g/L tryptone, 125 µL/L micro-mineral solution, 250 mL/macro-mineral solution, 250 mL/L reducing solution, and 1.25 mL/L of a 1 g/L resazurin solution prepared in distilled water were added in strict order. The micromineral solution contained 132 g/L CaCl_2_(H_2_O)_2_, 100 g/L MnCl_2_(H_2_O)_4_, 10 g/L CoCl_2_(H_2_O)_6_, and 80 g/L FeCl_3_(H_2_O)_6_ (Sigma-Aldrich). The micro-mineral solution was prepared in a 4 g/L (NH_4_)HCO_3_ buffer solution and 35 g/L NaHCO_3_ (Sigma-Aldrich). The macro-mineral solution contained 5.7 g/L Na_2_HPO_4_, 6.2 g/L KH_2_PO_4_, and 0.6 g/L MgSO_4_(H_2_O)_7_ in distilled water. The reducing solution was prepared immediately before use and consisted of 6.25 g/L anhydrous L-cysteine, 6.25 g/L Na_2_S(H_2_O)_9_, and 40 mL/L of 1M NaOH (Sigma-Aldrich). The resazurin solution is a pH indicator, so the final fermentation medium was blue.

The fermentation medium was placed in serum-type reaction vials (Supleco, Bellefonte, PA, USA) with a rubber septum. Each vial had two outlets to which three-way valves were attached to bubble CO_2_ through one and add the fermentation medium through the other. First, 8.33 mL of reducing solution was added to each serum vial and placed in a water bath at 100 °C for 15 min with the valves open. CO_2_ was then bubbled into the fermentation medium through one of the valves until the medium turned pink (reduced).

Bacterial inoculum: Human gut bacteria were obtained from the feces of six adult volunteers (25–30 years old). The volunteers were well defined as “apparently healthy” by a physician and a nutritionist. The volunteers were of average weight (body mass index between 18.5 and 22.9), were not currently receiving any pharmacological or nutritional treatment, had not changed bowel habits, and had not taken any antibiotics in the month preceding the fecal donation.

The donors collected their feces and transferred them to CO_2_-filled bags to avoid contamination with other types of body fluid (blood or urine). The feces were exposed to CO_2_ immediately upon arrival at the laboratory, and equal proportions of feces from each donor were mixed and homogenized according to the recommendation for pooled feces (Aguirre et al., 2014) [71]. The pooled feces were homogenized with the sterilized fermentation medium at a ratio of 10% (fw/v) for 3 min. The mixture was then filtered (1 mm mesh) before immediate use. The filtrate was considered to be a bacterial inoculum.

Fermentation: 100 mg (dw) samples of the *AS*, PPJ, and beverage (substrates) were placed in serum vials (50 mL capacity, Supleco) with 8 mL of fermentation medium and 2 mL of inoculum. The vials were sealed, CO_2_ was bubbled, and the valves were closed and placed in a 37 °C shaking bath at 100 rpm (Dual Action Shaker and Water bath, PolyScience, Niles, IL, USA).

#### Quantification of Fermentation By-Products

The AS, PPJ, and beverage fermentation kinetics were monitored at 0, 2, 4, 8, 12, and 24 h. Lactulose was used as a positive control, and the vials of the fermentation medium and inoculum without a substrate were used as negative controls.

Gas production: Syringes of different volumes (1, 3, and 10 mL) were fitted to the three-way valve; the valve was opened slowly, and the displacement of the plunger was recorded.

pH variations: The rubber septum was removed, the fermented contents were transferred to centrifuge tubes, and the pH of the fermentation medium was measured (HI 2210 pH Meter, Hanna Instruments, Carrollton, TX, USA). To determine the rate of fermentation according to the decrease in pH, the pH values at different fermentation times were fitted to a linear regression model (pH/time) with the data of the first 8 h.

Sample preparation: After measuring the pH, the fermentation was stopped by adding 2.5 mL of 1 M NaOH to the centrifuge tubes with the fermented substrates of the AS, PPJ, and beverage. The samples were centrifuged at 2500× *g* (Z 36 HK centrifuge, rotor 220.78 V21, Hermle Labortechnik, Wehingen, BW, Germany) for 10 min at 4 °C and filtered through 0.45 μm cellulose filters (Thermo Fisher Scientific, Waltham, MA, USA) to obtain at least 3 mL of supernatant. In microcentrifuge tubes, 400 μL of filtered supernatant was mixed with 100 μL of internal standard (50 μmol/mL 2-methylvaleric acid solution [Sigma-Aldrich]), 10 μL of formic acid (Sigma-Aldrich), and 490 μL of Milli-Q water, and they were centrifuged again at 12,000× *g* for 15 min at 4 °C (rotor 221.28 V21, Z 36 HK centrifuge). After centrifugation, 700 μL of the supernatant was placed in chromatograph vials (Perkin-Elmer, Waltham, MA, USA).

SCFA quantification was performed following with a gas chromatograph Clarus 500/580 (Perkin-Elmer) equipped with a capillary column CARBOWAX (30 m, capillary 25.0 µm × 0.32 mm) and a flame ionization detector [87]. The detector temperature started at 95 °C, was maintained for 2 min, and then was increased to 180 °C at a rate of 20 °C/min. Helium was the mobile phase at a flow rate of 1 mL/min; the initial temperature of 95 °C was maintained for 2 min and then raised at 10 °C/min up to 250 °C. The quantification was carried out by interpolating standard curves previously prepared with each of the selected standards with five calibration points of 1.0, 2.5, 5.0, 10.0, and 20.0 µmol/mL (Supplementary Material S1).

### 4.7. Statistical Analysis

The results are presented as mean ± SD (standard deviation), n = 3. A one-way analysis of variance and Tukey’s multiple comparison tests were applied with a significance level of <0.05 when differences between groups were found. The OpenLab software (version 2.8) was used to extract the chromatograms and find the area values under each chromatogram’s curve. The SIGMAPLOT software Version 12 was used to calculate the area under the hydrolysis curve. The correlation between the glycemic index, the content of bioactive compounds, and the antioxidant capacity determinations were obtained using the Pearson correlation test (*p* < 0.01). The slopes of the linearization of the pH variation were compared with one another, and the departure from linearity was evaluated. The Prism^®^ software version 8 (GraphPad Software, La Jolla, CA, USA) was used for data visualization and statistical treatment.

## 5. Conclusions

This study demonstrates that a beverage made from a combination of Agave sap (AS) and prickly pear juice (PPJ) exhibits antioxidant properties through various mechanisms, making it a potential natural option for managing oxidative stress in chronic diseases. The presence of bioactive compounds in the beverage shows a direct relationship with its antioxidant capacity. Additionally, the beverage has a low glycemic index and glycemic regulatory properties. Although the fermentation of PPJ alone produces lower short-chain fatty acid (SCFA) concentrations, combining it with AS creates a complementary effect. This combination leads to lower gas volumes than AS alone and higher total SCFA concentrations than PPJ alone. Excessive gas production is a common undesirable side effect of fermentable fiber dietary interventions, and this combination can effectively mitigate this issue. This combination of ingredients creates a non-fermented fruit beverage with fermentable fiber, consistent with the all-natural and minimally processed functional food trends. Future studies should focus on direct comparative analyses and additional experiments to further elucidate the unique benefits of this combined beverage in in vivo models.

## Figures and Tables

**Table 1:** Composition of the *Agave* sap (AS), prickly pear juice (PPJ), and beverage.

Component (g/100 mL)	AS	PPJ	Beverage
Moisture	90.04 ± 0.11 ^a^	87.53 ± 0.08 ^c^	89.80 ± 0.09 ^b^
Ash	0.13 ± 0.03 ^a^	0.30 ± 0.06 ^a^	0.18 ± 0.04 ^a^
Protein	0.25 ± 0.25 ^a^	0.21 ± 0.15 ^a^	0.26 ± 0.18 ^a^

Mean values of three replicates ± SD. Different letters in a row indicate significant statistical differences according to Tukey’s multiple comparison tests, with a significance level of 0.05.

**Table 2:** Quantification of the free carbohydrates in the *Agave* sap (AS), prickly pear juice (PPJ), and beverage using HPLC.

Carbohydrate (mg/100 mL)	AS	PPJ	Beverage
Nystose	78.52 ± 3.93 ^a^	52.57 ± 1.88 ^b^	72.98 ± 3.83 ^a^
Kestose	44.8 ± 2.24 ^a^	33.45 ± 1.67 ^c^	39.26 ± 1.96 ^b^
Sucrose	74.43 ± 2.62 ^a^	71.51 ± 3.29 ^a^	70.08 ± 3.50 ^a^
Glucose	43.46 ± 2.17 ^c^	226.27 ± 4.09 ^a^	142.15 ± 5.67 ^b^
Fructose	191.43 ± 9.65 ^a^	103.26 ± 2.36 ^c^	158.16 ± 8.86 ^b^
Galacturonic acid	113.08 ± 6.43 ^c^	411.96 ± 1.91 ^a^	217.74 ± 13.46 ^b^
Mannose	238.54 ± 13.63 ^a^	-	151.28 ± 2.04 ^b^
Galactose	-	192.24 ± 4.63 ^a^	100.85 ± 1.15 ^b^
Rhamnose	222.53 + 10.87 ^b^	274.09 ± 4.84 ^a^	227.00 ± 1.58 ^b^
Total quantified	1006.79 ± 25.29 ^c^	1365.35 ± 29.51 ^a^	1179.50 ± 77.84 ^b^

Mean values of three replicates ± SD. Different letters in a row indicate significant statistical differences according to Tukey’s multiple comparison tests, with a significance level of 0.05.

**Table 3:** Content of the bioactive compounds in the *Agave* sap (AS), prickly pear juice (PPJ), and beverage.

	AS	PPJ	Beverage
TPC (GAE)	22.95 ± 1.29 ^c^	62.415 ± 0.24 ^a^	48.179 ± 0.42 ^b^
Flavonoids (QE)	1.33 ± 0.18 ^c^	15.92 ± 1.85 ^a^	7.52 ± 0.10 ^b^
Catechin (mg/100 mL)	30.00 ± 1.40 ^c^	63.53 ± 3.20 ^a^	46.05 ± 2.16 ^b^
Protocatechuic acid (mg/100 mL)	33.89 ± 0.36 ^c^	84.43 ± 0.75 ^a^	55.03 ± 0.39 ^b^
Gallic acid (mg/100 mL)	1.31 ± 0.09	1.23 ± 0.02 ^a^	1.58 ± 0.05 ^a^
Myricetin (mg/100 mL)	10.28 ± 4.86 ^a^	-	4.70 ± 1.20 ^a^
Total quantified (mg/100 mL)	79.97 ± 6.79 ^c^	153.78 ± 4.1 ^a^	112.56 ± 3.88 ^b^
Ascorbic acid (mg/100 mL)	4.49 ± 0.08 ^a^	4.59 ± 0.13 ^a^	5.20 ± 0.08 ^a^

Total bioactive compounds are expressed as mg gallic acid equivalents (GAE) per 100 mL, while flavonoids are expressed as mg quercetin equivalents (QE) per 100 mL. The mean values of three replicates were calculated ± SD. Different letters in a row indicate significant statistical differences according to Tukey’s multiple comparison tests, with a significance level of 0.05.

**Table 4:** Antioxidant and iron chelation capacity of the *Agave* sap (AS), prickly pear juice (PPJ), and beverage.

Method	AS	PPJ	Beverage
PFRAP (%)	96.05 ± 0.26 ^c^	88.04 ± 0.99 ^a^	95.29 ± 0.11 ^b^
SASA (%)	10.34 ± 0.16 ^c^	15.63 ± 0.52 ^a^	13.47 ± 0.30 ^b^
ABTS (TE)	873.33 ± 70.24 ^c^	2853.33 ± 23.01 ^a^	2000.00 ± 55.34 ^b^

Values reported as percent antioxidant capacity (%) of each test using 0.5 M gallic acid as standard for PFRAP and SASA methods and in mmol Trolox equivalents/mL (TE) of sample for the ABTS method. Mean values of three replicates ± SD. Different letters in a row indicate significant statistical differences according to Tukey’s multiple comparison tests, with a significance level of 0.05.

**Table 5:** Estimated glycemic index, α-amylase, and α-glucosidase inhibition activity of the *Agave* sap (AS), prickly pear juice (PPJ), and beverage.

	Glycemic index		
	AS	PPJ	Beverage
	37.35 ± 0.42 ^a^	30.04 ± 0.44 ^b^	26.39 ± 0.19 ^c^
	Enzymatic inhibition (%)		
α-amylase	-	NA	-
α-glucosidase	93.92 ± 6.44 ^b^	NA	95.56 ± 3.09 ^a^

Mean values of three replicates ± SD. NA = no activity. Different letters in a row indicate significant statistical differences according to Tukey’s multiple comparison tests, with a significance level of 0.05.

## Data Availability

Data will be made available upon request.

## Supplementary Materials

The following supporting information can be downloaded at: https://www.mdpi.com/article/10.3390/molecules29122742/s1, Supplementary Table S1: Correlation analysis; Supplementary Material S1: Standard curves for chromatographic identification and quantification; Supplementary Figure S1: Gas production during in vitro gut fermentation in positive (lactulose) and negative controls (blank); Supplementary Figure S2: pH changes during in vitro gut fermentation in positive (lactulose) and negative controls (blank); Supplementary Figure S3: SCFA production during the in vitro fermentation process of positive and negative controls. Values are presented as means ± standard deviation. Supplementary Table S2: Total production of SCFAs (µmol/mL) and molar ratios (% of total SCFAs produced) after 24 h of in vitro gut fermentation.
