# Supplementary material for: Health-Related Composition and Bioactivity of an Agave Sap/Prickly Pear Juice Beverage
Source: Molecules. 2024 Jun 8;29(12):2742. doi: 10.3390/molecules29122742 (PMC11206587; doi:10.3390/molecules29122742)
Supplement: Supplementary file 1 [file molecules-29-02742-s001.zip › molecules-3021231-supplementary.pdf]

Table S1. Correlation analysis of the content of bioactive compounds, antioxidant activity, and glycemic index of *Agave* sap, prickly pear juice, and the beverage

	**1**	**2**	**3**	**4**	**5**	**6**	**7**	**8**	**9**	**10**	**11**
**1. TPC**	**1.000**										
**2. Flavonoids**	**0.952**	**1.000**									
**3. PFRAP**	**0.820**	**0.918**	**1.000**								
**4. SASA**	**0.988**	**0.973**	**0.831**	**1.000**							
**5. ABTS**	**0.974**	**0.932**	**0.855**	**0.966**	**1.000**						
**6. GI**	- 0.765	**- 0.562**	**- 0.274**	- 0.721	- 0.704	**1.000**					
**7. Ascorbic acid**	**- 0.940**	**- 0.808**	**- 0.581**	**- 0.911**	**- 0.890**	**0.938**	**1.000**				
**8. Catechin**	**- 0.895**	- 0.739	**- 0.493**	**- 0.859**	**- 0.843**	**0.968**	**0.992**	**1.000**			
**9. Gallic acid**	**- 0.882**	- 0.719	**- 0.461**	**- 0.847**	**- 0.825**	**0.978**	**0.990**	**0.997**	**1.000**		
**10. Protocatechuic acid**	**- 0.939**	**- 0.813**	**- 0.589**	**- 0.914**	**- 0.897**	**0.932**	**0.994**	**0.986**	**0.984**	**1.000**	
**11. Myricetin**	- 0.558	- 0.740	**- 0.913**	- 0.592	- 0.607	- 0.095	0.251	0.155	0.111	0.256	**1**

Bold numbers represent a significant correlation at 0.01. TPC: total phenolic compounds, PFRAP: Potassium Ferricyanide Reducing Power Assay; SASA: Superoxide anion scavenging activity test; ABTS: total radical scavenging activity.

Table S2. Total production of SCFA (µmol/mL) and molar ratios (% of total SCFA produced) after 24 hours of *in vitro* intestinal fermentation.

Substrate	AS		PPJ		B	
	Total production	Molar ratio	Total production	Molar ratio	Total production	Molar ratio
Acetate	16.77±1.60$_a$	45.22±4.33$_b$	16.36±1.02$_a$	60.29±3.77$_a$	16.96±1.26$_a$	56.11±4.18$_a$
Propionate	4.92±0.76$_a$	13.27±2.05$_a$	3.92±0.28$_a$	14.45±1.04$_a$	4.36±0.39$_a$	14.42±1.30$_a$
Butyrate	12.47±0.77$_a$	33.62±2.08$_a$	6.85±0.87$_b$	25.26±3.22$_b$	8.91±0.89$_b$	29.47±2.94$_b$
Valerate	2.93±0.23	7.89±0.61	-	-	-	-
Total	37.09±3.36	100	27.14±2.18	100	30.23±2.54	100

Presented values are means ± standard deviation from two biological replicates (n = 3). Values with different letters within each SCFA are significantly different from each other, comparing total production and molar ratio independently.

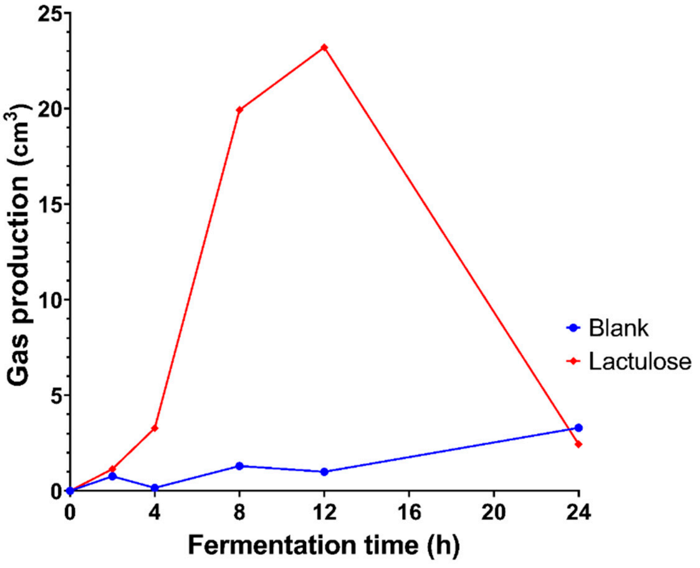

Figure S1 Gas production during *in vitro* colonic fermentation in positive (lactulose) and negative control (blank).

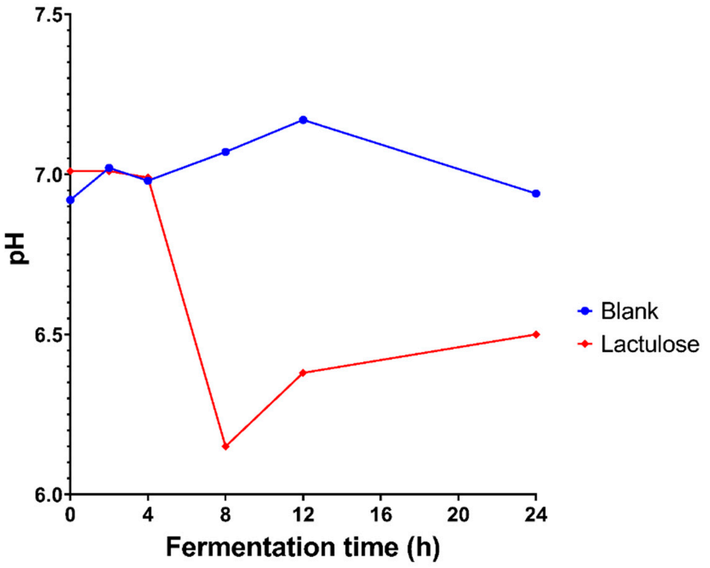

Figure S2 pH changes during *in vitro* colonic fermentation in positive (lactulose) and negative control (blank).

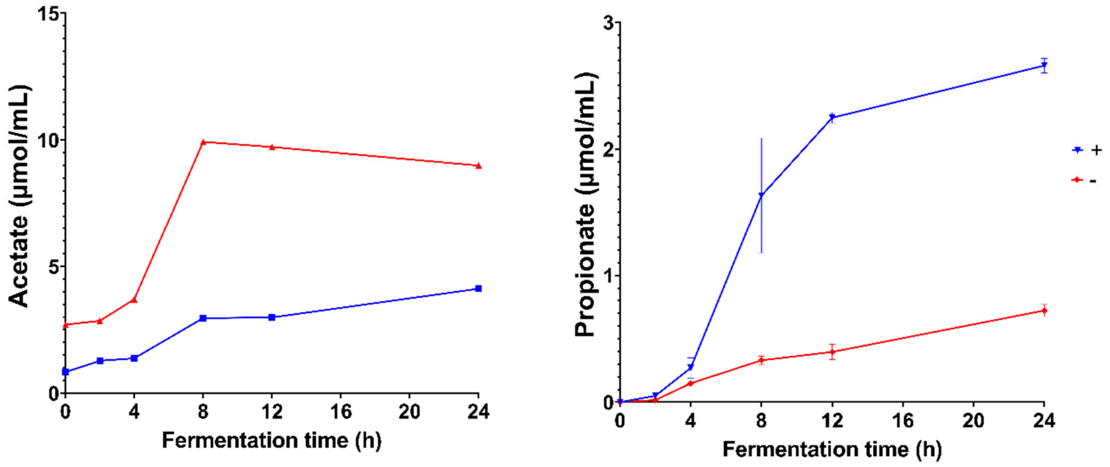

Figure S3 SCFA production during the *in vitro* fermentation process of positive and negative control. Values are means ± standard deviation.

Supplementary material S1.

Standard curves for individual carbohydrates identification and quantification

- Nystose (2.00, 3.00, and 4.00 mg/ml; y=28288430.25x-10401275.75; R^2=0.98)
- Kestose (0.30, 4.00, and 4.00 mg/ml; y=16,482,480.84x+4,192,383.57; R^2=1.00)
- Sucrose (1, 5, 10, 20, and 30.00 mg / ml; y=22,954,952.46x-6,117,608.90; R^2=1.00)
- Glucose (1, 5, 10, 20, and 30.00 mg / ml; y=23714768.52x+23439121.49 R^2=0.99)
- Fructose (1, 5, 10, 20, and 30.00 mg / ml; y=20953623.53x+21983163.82; R^2=1.00)
- Glucuronic acid (20, 15, 10, and 5 mg / ml; y=38394690.45x+695733.76; R^2=0.999)
- Galacturonic acid (30, 20, 10, 5, and 1 mg / ml; y=37260683.60x+7973218.96; R^2=0.999)
- Mannose (30, 20, 10, 5, and 1 mg / ml; y=41293843.57x+4594900.44; R^2=0.999)
- Galactose (30, 20, 10, 5, and 1 mg / ml; y=42599665.93x+3118747.94; R^2=0.999)
- Xylose (30, 20, 10, 5, y 1 mg / ml; y=40469795.17x+6340066.0; R^2=0.999)
- Rhamnose (30, 20, 10, 5, y 1 mg / ml; y=38455572.16x+6772687.15; R^2=0.999).

Standard curves for individual bioactive compounds identification and quantification

- Rutin (0.0039, 0.007, 0.031, 0.062, and 0.125 mg/ml; y=1E+07x+2881.1; R^2=0.999)
- Gallic acid (0.015, 0.03, 0.06, 0.12, and 0.25 mg/ml; y=4376855.87x+67727.38; R^2=0.999)
- Catechin (0.015, 0.031, 0.062, and 0.25 mg/ml; y=962685x+5382.2; R^2=0.999)
- Caffeic acid (0.015, 0.031, 0.062, and 0.25 mg/ml; y=4E+07x-30040; R^2=0.999)
- Quercetin (0.031, 0.062, 0.125, 0.25, and 0.5 mg/ml; y=3E+07x-8571; R^2=0.999)
- P–coumaric acid (0.031, 0.062, 0.125, 0.25, and 0.5 mg/ml; y=6E+07x+410249; R^2=0.999)
- Pelargonidin (0.031, 0.062, 0.125, 0.25, and 0.5 mg/ml; y=4E+07x-1E+06; R^2=0.999)
- Ferulic acid (0.031, 0.062, 0.125, 0.25, and 0.5 mg/ml; y=7E+07x-594982; R^2=0.999)
- Protocatechuic acid (0.03, 0.06, 0.12, and 0.25 mg/ml; y=5E+07x-118957; R^2=0.989)
- Ascorbic acid (0.05, 0.1, 0.2, 0.4, 0.6, and 0.8 mg/ml, y=2E+07x-162364; R^2=0.997)
- Myricetin (y=858141x-1337.3; R^2=0.999)
- Chlorogenic acid (0.05, 0.1, 0.4, 0.6, 0.8, and 1 mg/ml; y=2E+07x+284103; R^2=0.999).

Standard curves for individual short-chain fatty acids identification and quantification

- Acetic acid (y=558.13x-207.1; R^2=0.99)
- Propionic acid (y=942.69x+109.55; R^2=0.99)
- Isobutyric acid (y=1194.6x+313.32; R^2=0.99)
- Butyric acid (y=1368.6x-382.68; R^2=0.99)
- Isovaleric acid (y=834.19x+383.66; R^2=0.99)
- Valeric acid (y=977.85x+736.65; R^2=0.99).
